# Eccentric Training in Pulmonary Rehabilitation of Post-COVID-19 Patients: An Alternative for Improving the Functional Capacity, Inflammation, and Oxidative Stress

**DOI:** 10.3390/biology11101446

**Published:** 2022-10-01

**Authors:** Felipe Contreras-Briceño, Maximiliano Espinosa-Ramírez, Dmitry Rozenberg, W. Darlene Reid

**Affiliations:** 1 Laboratory of Exercise Physiology, Department of Health Science, Faculty of Medicine, Pontificia Universidad Católica de Chile, Av. Vicuña Mackenna #4860, Santiago 7820436, Chile; 2 Physiology Section, Department of Cell Biology, Physiology and Immunology, Faculty of Biology, Universitat de Barcelona, 08028 Barcelona, Spain; 3 Advanced Center for Chronic Diseases (ACCDiS), Division of Cardiovascular Diseases, Facultad de Medicina, Pontificia Universidad Católica de Chile, Marcoleta #367, Santiago 8380000, Chile; 4 Millennium Institute for Intelligent Healthcare Engineering, Av. Vicuña Mackenna #4860, Santiago 7820436, Chile; 5 Department of Medicine, Respirology, University of Toronto, Toronto, ON M5G 2C4, Canada; 6 Toronto General Hospital, Research Institute, University Health Network, Toronto, ON M5G 2C4, Canada; 7 Department of Physical Therapy and Interdepartmental Division of Critical Care Medicine, University of Toronto, Toronto, ON M5G 2C4, Canada; 8 KITE Research Institute, Toronto Rehabilitation Institute, University Health Network, Toronto, ON M5G 2A2, Canada

**Keywords:** post-COVID-19, exercise, physical activity, rehabilitation

## Abstract

**Simple Summary:**

Exercise is the cornerstone of pulmonary rehabilitation (PR) programs. Although all exercise has concentric (CONC, where the muscle groups shorten to produce force) and eccentric (ECC, characterized by lengthening muscle during its muscle contraction) contractions or components, usually PR is focus on CONC as the main training modality. Data show that this exercise modality increases cardiopulmonary stress, eliciting higher ventilation and consequently exacerbating dyspnoea, been more stressful for patients with chronic respiratory diseases (e.g., COPD and interstitial lung diseases). Similarly, higher cardiopulmonary stress has been associated with more oxidative stress. This aspect has been proposed as the central pathophysiological mechanism involved in the functional limitation of post-COVID-19 patients with moderate-to-severe damage associated with SARS-CoV-2 infection and prolonged hospital stay. Thus, traditional PR programs with CONC as a primary exercise modality can exacerbate dyspnoea and leg fatigue in these patients, repercussing in early exercise intolerance and diminishing the adherence to PR. On the other hand, ECC training has increased the locomotor muscle mass without significant cardiopulmonary stress, improving functional capacity and self-autonomy. This review discusses the mechanism that supports aerobic ECC exercise as a novel alternative to conventional CONC exercise included in traditional PR for post-COVID-19 patients.

**Abstract:**

The purpose of this narrative review is to highlight the oxidative stress induced in COVID-19 patients (SARS-CoV-2 infection), describe longstanding functional impairments, and provide the pathophysiologic rationale that supports aerobic eccentric (ECC) exercise as a novel alternative to conventional concentric (CONC) exercise for post-COVID-19 patients. Patients who recovered from moderate-to-severe COVID-19 respiratory distress demonstrate long–term functional impairment. During the acute phase, SARS-CoV-2 induces the generation of reactive oxygen species that can be amplified to a “cytokine storm”. The resultant inflammatory and oxidative stress process causes organ damage, particularly in the respiratory system, with the lungs as the tissues most susceptible to injury. The acute illness often requires a long-term hospital stay and consequent sarcopenia. Upon discharge, muscle weakness compounded by limited lung and cardiac function is often accompanied by dyspnea, myalgia, anxiety, depression, and sleep disturbance. Consequently, these patients could benefit from pulmonary rehabilitation (PR), with exercise as a critical intervention (including sessions of strength and endurance or aerobic exercises). Unfortunately, conventional CONC exercises induce significant cardiopulmonary stress and increase inflammatory and oxidative stress (OS) when performed at moderate/high intensity, which can exacerbate debilitating dyspnoea and muscle fatigue post-COVID-19. Eccentric training (ECC) is a well–tolerated alternative that improves muscle mass while mitigating cardiopulmonary stress in patients with COPD and other chronic diseases. Similar benefits could be realized in post-COVID-19 patients. Consequently, these patients could benefit from PR with exercise as a critical intervention.

## 1. Introduction

The coronavirus disease 2019 (COVID-19) pandemic has significantly increased the number of patients hospitalized for pneumonia and acute respiratory distress syndrome (ARDS) [1]. COVID-19, caused by the novel severe acute respiratory syndrome coronavirus 2 (SARS-CoV-2), mainly attacks cells in the respiratory system [2,3]. In the acute phase of COVID-19, patients with moderate–to–severe ARDS are characterized by an elevated pro-inflammatory state secondary to a “cytokine storm” (CS) [4,5,6,7]. This process stimulates the generation of reactive chemical species (RS) and induces oxidative stress (OS) [8,9,10], that has been postulated as the primary cause of tissue damage and consequent functional impairments post-COVID-19 [10,11,12]. Fatigue and muscle weakness are the primary impairments reported in patients post-COVID-19 even six months after medical discharge [13]. These prolonged sequalae underscores the longstanding impact of the heightened inflammatory process and OS, muscle damage and sarcopenia secondary to viral infection, and the prolonged hospital stay. 

Exercise is the cornerstone of pulmonary rehabilitation (PR) programs that is essential to restore self–autonomy and the ability to perform meaningful activities of daily living in these patients (including sessions of strength and endurance or aerobic exercises). Regarding aerobic exercise, the type and intensity of training should be adequately prescribed by the health professional to be well tolerated by patients and achieve the high adherence required to reverse the physical impairments. The prescribed exercise needs to promote muscle’s function and mass while minimizing cardiopulmonary stress. The mainstay exercise in PR is concentric (CONC) in nature, which unfortunately stimulates significant cardiopulmonary stress to reach the exercise intensities that induce clinical improvements. In contrast, eccentric (ECC) exercises, when muscles contract while lengthening, improve muscle function and mass with minimum cardiopulmonary stress; this is a relative novel mode of clinical training despite its high requirement during daily activities (e.g., walking downstairs or hills, sitting down in a chair). Substantial evidence indicates that ECC compared to CONC training induces improvement in functional capacity with less dyspnoea and fatigue [14,15,16]. Whether these benefits occur in patients with moderate–to–severe damage by COVID-19 is not known.

This narrative review highlights the oxidative stress induced in COVID-19 patients, describe longstanding functional impairments, and provides the pathophysiologic rationale that supports aerobic ECC exercises as an alternative to conventional CONC exercises, including in traditional pulmonary rehabilitation programs for patients with moderate–to–severe damage recovered from COVID-19.

## 2. Epidemiological Data about COVID-19 and Exercise as a Therapeutic Intervention

At the middle of September 2022, COVID-19 has affected more than 610 million cases worldwide, with near to 6.50 million confirmed deaths [17]. The epidemiology of COVID-19 infection has evolved over the last 2 years with availability of vaccinations, pharmacotherapy, and our understanding of pathogenesis [18,19]. Early during the pandemic with the prevailing Delta strains and lack of availability of vaccinations, approximately 20% of the diagnosed cases require hospitalization due to the severity of the symptoms [20], with 14.0 (10.0–19.0) days as the median hospitalization time [21]. Of those admitted, between 17–35% require support in critical care units, mainly due to hypoxemic respiratory failure [22,23,24], which necessitates a more prolonged hospital stay. However, recent evidence suggests that moderate or severe disease has been less likely during the period with Omicron compared to Delta strain; however, common risk factors for hospitalization or admission to critical care have included older age, immunosuppression, and comorbidities [25,26]

Admission to hospital or critical care with SARS-CoV-2 infection can have important deleterious consequences from bed rest, critical illness polyneuromyopathy syndrome (CIPNM, 10–30% of prevalence) [27,28,29], loss of strength, dyspnoea, and multiorgan involvement (liver, myocarditis, and brain damage) [23,30]. At medical discharge, significant numbers of these patients experience persistent dyspnoea, anxiety, depression, chest pain, fatigue, and muscle weakness. When these symptoms persist for 3 months or longer, cases are designated “post-COVID-19 syndrome” [31,32] and impact self-autonomy and mortality. Based on the epidemiological data together with functional impairments of patients with post COVID-19 condition, PR has a crucial role in improving the functional capacity and health–related quality of life (HRQoL) by providing effective and well–tolerated training methods to achieve the required high adherence to induce adequate physiological adaptation [33,34].

## 3. Role of Inflammatory and OS Markers in COVID-19

The cellular entry of SARS-CoV-2 depends on the viral structural spike protein binding to angiotensin-converting enzyme 2 (ACE2) receptor, and the fusion of membranes mediated by the type 2 transmembrane serine protease (TMPRSS2) of the host cells [2,35]. The ACE2 receptor and TMPRSS2 are expressed mainly in the respiratory system, but also by the intestine, kidneys, and heart [36,37]. The lungs are the main target organ of SARS-CoV-2, due to their large surface area exposed inhaled infectious agents and the nature of type II epithelial cells [38]. Inside the cell, SARS-CoV-2 releases their RNA, and more virions are able to replicate. When the virus is recognized, macrophages and neutrophils are recruited to the infection site, initiating an overproduction of cytokines and consequent CS [6,7]. Moreover, interleukin–1β (IL–1β), IL–6, IL–10, interferon-gamma (IFN–γ), and tumor necrosis factor-alpha (TNF–α) are the major inflammatory markers elevated in patients infected by SARS-CoV-2 contributing to the CS [39,40,41,42,43].

Cytokines are proteins that act as signaling molecules that recruit immune cells to the site of inflammation, induce vascular leakage and exudation, and stimulate the formation of reactive species (RS) to eliminate the virus, promoting OS. The OS is defined as an imbalance between RS and antioxidants in favor of the RS, leading to a disruption of redox signaling and control, and/or molecular damage [44]. The evidence suggests that the overproduction of RS and/or a low quantity of antioxidants is associated with the pathogenesis, progression, severity, and sequelae induced by SARS-CoV-2 infection [45,46]. To understand the origin of the dysfunctions seen in patients with COVID-19, it is necessary to recognize the role of RS as the factors executing tissue damage and organ deterioration.

## 4. Redox Imbalance in Patients with COVID-19

**Figure 1:**
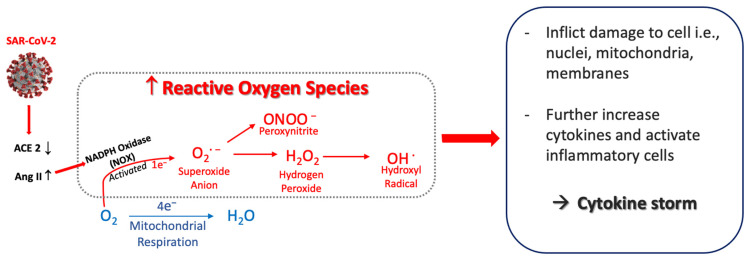
Postulated sequence that generates reactive oxygen species from SARS-CoV-2. SARS-CoV-2 infection induces a decrease in ACE2 that causes Ang II to increase, which activates NADPH oxidase (NOX). This generates superoxide anion (O_2_^·−^) and other oxygen radicals—peroxynitrite, hydrogen peroxide and hydroxyl radical. The reactive oxygen species inflict cellular damage, further activate inflammatory cells, and amplify the increased release of cytokines. **Abbreviations:** ACE2, angiotensin–converting enzyme 2; Ang II, angiotensin II.

The RS are produced by reactions during respiration and can be generated by phagocytic cells including neutrophils and macrophages [47]. An overview of the main RS is shown in Figure 1.

In viral infections, a major main source of RS production is the nicotinamide adenine dinucleotide phosphate (NADPH) oxidases (NOXs), the main enzymes expressed by macrophages [48]. In COVID-19, NOXs have been recognized as the main source of RS formation. The binding of SARS-CoV-2 to the ACE2 receptor results in increases in angiotensin II (Ang II) because the ACE2 receptor is no longer available to convert Ang II to Ang 1–7 (renin-angiotensin-aldosterone system, RAAS). Subsequently, Ang II binds the angiotensin type 1 receptor (AT1R) and stimulates NOXs activity. NOXs act by reducing O_2_ to superoxide anion (O_2_^−^) [49] leading to overproduction of O_2_^−^ [50], which in turn can initiate production of other oxygen radicals.

The O_2_^−^ can react with other reactive species to produce hydrogen peroxide (H_2_O_2_) [51]. H_2_O_2_ is relatively stable with a prolonged half-life so often measured at systemic or respiratory levels as marker of RS. Nonetheless, it is considered cytotoxic at high concentrations because it can generate the very harmful RS, the hydroxyl radical (OH^−^). OH^−^ can damage inorganic and organic molecules, including lipids, proteins, carbohydrates, and DNA. It has a short half–life, and its high reactivity allows it to damage molecules very close to its formation site. Due to its reactivity, it is impossible to determine its quantity or concentration, so it is common to evaluate the damage associated with their production [52], such as derivates of arachidonic acids oxidation and prostaglandins (e.g., 8–isoprostane, 8–iso–PGF2α) [53].

Another source of RS production implicated in viral infections is inducible nitric oxide synthase (iNOS) enzyme present in macrophages and neutrophils [54,55]. iNOS rapidly produces high levels of nitric oxide (NO^·^), and their derivates nitrite (NO_2_^−^) and nitrate (NO_3_^−^) for reducing pathogens [56]. The NO^·^ in the presence of O_2_^−^ from NOXs reactions can produce peroxynitrite (ONNO^−^), a highly harmful RS that generates lipid peroxidation of biological membranes and 8–iso–PGF2α formation [53]. Add to this RS increases, in viral infections have been found the inhibition of pathways mediated by the nuclear factor erythroid related factor 2 (Nrf2), which is a master transcription regulator of genes related to antioxidant enzymes necessaries to counteract the increases of O_2_^−^ and H_2_O_2_ (superoxide dismutases (SODs), glutathione peroxidases (GPXs), and catalase (CAT)) [57,58]. Concerning, have been reported a direct association between low expression of SOD and disease severity in lungs of elderly patients with COVID-19 [59].

It is considered that OS in at risk COVID-19 patients is due to an excess of RS that is not countered by an increase in antioxidants. OS levels in these patients have been determined from an increase in inflammatory markers such as cytokines. However, this evidence is mainly supported by review articles. To our knowledge, only one study has evaluated in patients with COVID-19 inflammatory and OS markers (H_2_O_2_, malondialdehyde (MDA), and total antioxidant capacity (TAC)) by blood samples, finding no correlation with the severity of the disease [60]. Therefore, investigations are needed to document inflammatory and OS markers in COVID-19 patients to evaluate their association with tissue damage and consequent functional impairments in the acute phase of the disease. Future studies need to not only evaluate measures of their systemic effect (blood or urine samples) but also markers of the respiratory system (e.g., exhaled breath condensate (EBC) samples); the latter of which is essential to understand the primary target organ of SARS-CoV-2 and its greater susceptibility to tissue damage.

## 5. What Are the Main Sequelae Reported by Patients Recovered from COVID-19?

Patients recovered from COVID-19 differ in characteristics depending on whether they developed ARDS during hospitalization. Anastasio et al. [61] evaluated functional outcomes four months after diagnosis, finding that those who developed ARDS had lower total lung capacity and oxygen saturation (SpO_2_) at rest and more significant dyspnoea and decreased SpO_2_ during exercise (six–minute walking test, 6mWT). Emerging evidence is becoming available that reports the prevalence of symptoms, signs, and functional sequelae in the most severe patients recovered from SARS-CoV-2 infection. Upon discharge, they have decreased total lung capacity and lung diffusion [62,63,64], myocarditis, high blood pressure, arrhythmias [30], respiratory and locomotor muscles weakness [65,66], and critical illness myopathy syndrome [27]. Highly prevalent symptoms are: fatigue and muscle weakness (63–81%), dyspnoea (60%), myalgia (50%), sleep disturbances (27%), and anxiety and depression (23–32%).

**Figure 2:**
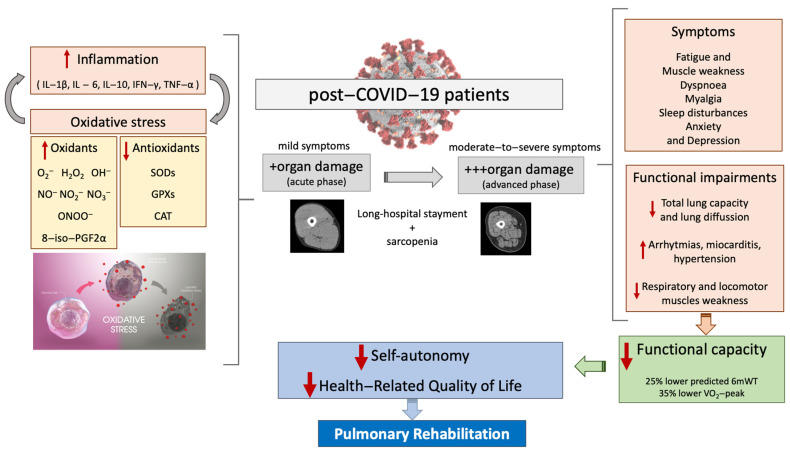
Overview of the factors involved in the deterioration of the functional capacity, self-autonomy, and health-related to quality of life in post-COVID-19 patients that need to be addressed by pulmonary rehabilitation. **Abbreviations**: IL = interleukin; IFN–γ = interferon gamma; TNF–α = tumor necrosis factor alpha; O_2_^−^ = anion superoxide; H_2_O_2_ = hydrogen peroxide; OH^−^ = hydroxyl ion; NO^−^ = nitric oxide; NO_2_^−^ = nitrite; NO_3_^−^ = nitrate; ONOO^−^ = peroxynitrite; 8–iso PGF2α = 8–iso–prostagladin F2–alpha; SOD = superoxide dismutase; GPX = glutathione peroxidase; CAT = catalase; 6mWT = six-minute walking test; VO_2_-peak = peak oxygen uptake.

All these factors can impact functional capacity as indicated by a 25% lower predicted 6mWT distance [67], and 35% lower predicted values of peak aerobic capacity (VO_2_-peak) [68]; impairments that decrease self–autonomy [69], and the HRQoL [70]. Follow–up at six–weeks after medical discharge in 33 patients revealed that despite no further decrease in lung function, patients reported fatigue (45%), dyspnoea (33%), cough (33%); lower 6mWT distance (−118 m of predicted value); and decreased HRQoL [71]. Six months after discharge, comparable findings were reported in 1733 patients; although restrictive ventilatory alterations were restored, these limitations remained (fatigue and muscle weakness, sleep–difficulties, and anxiety), including a 23% less 6mWT distance than the lower limit of the normal range (87.7 (75.9 to 101.1) of percentage of predicted value) [13]. These data strongly infer that functional capacity remains diminished despite improved lung function, suggesting that extrapulmonary factors, such as muscle function and mass, are involved in the physical impairments of patients recovered from COVID-19. Accordingly, the training mode in PR should be effective to restore the self-autonomy that enables an early social reintegration [72,73]. A summary of the topics discussed previously is presented in Figure 2.

## 6. Data about Pulmonary Rehabilitation in Patients Post-COVID-19?

A recent review reported that only three studies had evaluated the effect of PR in outpatients post-COVID-19. It concluded that PR could improve exercise capacity measured by 6mWT among patients with mild-to-moderate lung impairment (50.4 m, 95% CI 34.3 to 66.4 m). In contrast, the interpretation of effects on lung function, symptoms of dyspnoea and leg fatigue, and HRQoL was more cautious due to inadequate and conflicting data reported across studies [63]. The first published study appears to have methodological issues. This randomized control trial (RCT) by Liu et al. [74] demonstrated increases in lung function, 6mWT distance, and HRQoL in 36 patients after 12 sessions (6 weeks) of endurance exercises plus inspiratory muscle training (IMT); however, they did not provide details of training program (intensity, duration, type of exercise). Moreover, patients had relevant clinical variability which may prevent generalizability of these data. The second study (a pilot study) only performed IMT in patients after weaning from mechanical ventilation but did not include endurance training [75]. The third study by Li et al. [76] showed that an unsupervised home–based 6–weeks of aerobic exercise, thoracic expansion techniques, and lower limb muscle strength exercise increased the 6mWT distance (82 m), but not lung function, dyspnoea, and HRQoL. The aerobic exercises consisted of running (2 times daily, 40–60 min per session, 3–4 sessions by week plus teleconsultations once a week) with intensity based on heart rate reserve (from 30–40% initially, which progressed to 40–60% as tolerated) and Borg’s rating of perceived exertion (RPE) (from 11 to 14 score to 20). Although relevant results were obtained, its narrow inclusion criteria of patients with moderate dyspnoea symptoms limits generalizable to patients with moderate–to–severe damage by COVID-19, the main patient groups who require PR programs after medical discharge. The remaining articles related to PR in patients post-COVID-19 in this review were letters to the editor or cross–sectional studies.

In other coronavirus diseases (i.e., SARS-CoV-1 and MERS), few studies have evaluated the role of physical training. Lau et al. [77] studied 71 patients post-SARS-CoV-1 after 6 weeks of 30–45 min of cycling or running, 4–5 sessions by week. They concluded that training increased exercise capacity (77 m in 6MWT), with no effect on lung function, dyspnoea, leg fatigue and HRQoL. Another study by Zhang et al. [78] performed follow-up of 142 patients post-SARS-CoV-1. They found that the lung lesions induced by the virus recovered to a better extent in those patients whose completed a PR, but they did not give details about exercise training. Regarding patients post-MERS, we did not find evidence about the effect of exercise on functional variables.

To date, evidence supports that PR in patients recovered from coronavirus infection increase functional capacity. However, such changes have not necessarily translated towards improvement of activities of daily living and greater functional independence. Moreover, results were obtained come from patients with mild to moderate involvement. These findings limit generalizability to patients with moderate to severe damage who have more complex multisystemic effects of COVID-19 compounded by prolonged hospital stay and peripheral muscle damage that could more significantly affect functional capacity.

## 7. Effect of Aerobic Exercise/Training on Inflammatory and OS Markers

It is well described that acute exercise induces an increase in pro–inflammatory cytokines and OS markers [79,80,81], being more pronounced after bouts of ECC than CONC exercises [82,83]. Of interest, chronic CONC and ECC exercise not only leads to a reduction in these outcomes [84,85,86,87], but patients also benefit by increases in functional capacity, HRQoL, reduction in hospitalization, morbidity, and mortality. To date, there are not similar data from patients post-COVID-19 or recovered from others coronavirus infections (i.e., SARS-CoV-1 and MERS). Thus, evidence is lacking regarding the impact of the virus infection on biological response to physiological stress induced by exercise.

A commonly used protocol to evaluate the training-induced changes utilizes serial analyses of biological samples before and after the completion of an acute submaximal exercise; thus, to evaluate the adaptations induced by training, the changes of markers during the protocol are analyzed. This procedure mitigates interpretation errors associated with the intrinsic variability of markers commonly used in clinical studies. By this method, Mercken et al. [88] reported that 8-weeks of CONC exercise in 11 patients with moderate COPD decreased the hydrogen peroxide (H_2_O_2_) in EBC samples and malondialdehyde (MDA) in blood and increased the exercise capacity (VO_2_-peak and 6mWT). Similar results were reported by Rodriguez et al. [89] in 18 patients with severe COPD. Based on these results, it can be deduced that training decreases OS markers and increases functional capacity in respiratory diseases; however, it is not known if the effect differs depending on the training mode performed (ECC vs. CONC). The analysis of these markers can better inform how disease phenotype or respiratory conditions, with or without comorbidities, respond to different types of training.

Considering previous studies, ECC training has the potential to be a valuable alternative for patients with limited capacity to reach and sustain sufficient CONC exercise intensities to induce significant functional gains. To our knowledge, the physiologic response and benefit of ECC exercises in post-COVID-19 patients is unexplored. Thus, it is necessary to evaluate its clinical impact by comparing supervised ECC versus CONC training on functional capacity and related variables in outpatients with moderate-to-severe damage by COVID-19 to identify the most well tolerated and effective training method to apply in PR programs.

## 8. Aerobic Eccentric Training for Pulmonary Rehabilitation in Patients Post-COVID-19

Patients post-COVID-19 have reduced lower limb muscle mass and strength [90], specifically the knee extensors muscles (i.e., *quadriceps muscle*). This impairs functional capacity and self–autonomy, resulting in sedentary behaviors and exacerbating leg fatigue symptoms, muscle weakness during daily activities, and sarcopenia [91,92]. Thus, the training mode performed during PR programs should be well tolerated by patients and effective for improving their functional capacity, autonomy and HRQoL. The conventional aerobic exercises included in the standard of PR are cyclic movements, mainly focused on lower limbs (e.g., running, jogging, or walking (depending of the functional impairments), actions that implicate CONC and ECC contractions, but being the CONC the more predominantly where the treadmill is set up with positive slope) and cycling (with CONC as the primary movements)).

To effectively improve skeletal muscle performance, exercise training load must be greater than the regular daily physical activity [93]. Although CONC are commonly used and relatively safe, it induces a significant cardiovascular and respiratory stress when exercise is completed at moderate-to-severe intensity. Dyspnea followed by leg fatigue are primary patient reported outcomes for stopping exercise in chronic cardiorespiratory diseases [94,95,96]. In post-COVID-19 patients, it has been proposed that hypermetabolism, secondary to an increased catabolic process induced by SARS-CoV-2 infection [97], could further impede their ability to reach and sustain sufficiently high exercise intensities to promote the beneficial training–induced changes.

ECC contractions, when muscle lengthens while producing force, it is a relatively novel mode of training proposed to be potentially effective for patients with exercise intolerance [98,99,100,101]. ECC training can induce greater gains in muscle power while inducing lower metabolic and cardiorespiratory demands than CONC training (by 4 to 5 fold) [98,101,102]. ECC could be implemented during cycling or treadmill walking, using special ergometers. An ECC cycle ergometer utilizes an electrical motor that moves the cranks backwards and consequently is countered by ECC contractions mainly of the knee extensor muscle group [103].

ECC training appears to induce lesser cardiac and ventilatory stress than CONC training. Compared with CONC, the oxygen cost to be considerably lower (about 1/5 of CONC) [104], and, for a given level of VO_2_ (higher 1 L·min^−1^) cardiac output is higher and systolic volume lower during ECC training [104,105]. This enables patients to perform greater workloads of muscle power in a session than traditional CONC cycling training under comparable cardiorespiratory stress [106,107]. Regarding ventilatory stress induced by ECC versus CONC training, healthy subjects have demonstrated lower minute ventilation, tidal volume and hyperpnea at the same intensity [108].

Related to a lower cardiorespiratory stress, ECC training has demonstrated benefits in COPD patients. Ward et al. (2021) recently reported that ECC cycling was described to be more enjoyable than CONC by COPD patients. It was also associated with lower production of lactate and creatine kinase levels (markers of glycolysis and muscle damage, respectively) [109]. Moreover, MacMillan et al. (2017) reported that COPD patients were able to perform ECC cycling at a 3-fold higher workload with lower fatigue and dyspnoea during 10 weeks of training when compared to CONC cycling. Further, ECC cycling increased knee extensor strength by 16% and lower limb muscle mass by 2%, with no commensurate changes in the CONC cycling group [106]. Recently, Inostroza et al. (2022) reported that 34 sessions (30 min, 5–6 to RPE) in COPD patients produced a 3–fold greater workload, 1.5% higher SpO_2_, 24% lower hear rate (HR), 64% lower dyspnoea, and notably improved functional capacity (25% of 6mWT distance) than CONC training [15]. Camilo et al. (2015) reported in COPD patients that walking downhill showed lower minute ventilation and VO_2_ at a similar exercise intensity than CONC walking (9 and 10% less about peak values, respectively) [110]. Further, they found that 12–weeks of ECC training (−10% decline at treadmill) induced more COPD patients to achieve the 30 m of change at 6MWT than conventional CONC (94% vs. 65%), with a faster progression of treadmill speed and lower dyspnoea [111]. Taken together, ECC cycling, and walking induced considerable physiologic benefits and functional improvements while imposing lower degree of dyspnoea.

While ECC training can be a very effective training approach, it is essential to monitor potentially harmful outcomes that are more prevalent during the initial exercise sessions. ECC exercise can induce muscle injury manifested as cellular damage and plasma markers (e.g., creatine kinase levels). This injury can be accompanied by muscle weakness and delayed–onset muscle soreness (DOMS) [14,112]. Injury at the light microscopic or ultrastructural level is shown by disruption of contractile and structural proteins, increased permeability of plasma membranes and an influx of inflammatory cells [113,114]. However, when ECC exercises are repeated within weeks of the training period, changes in muscle damage markers are attenuated, and recovery is enhanced. This adaptation is called the *repeated bout effect* [115]. Another consideration is the increased susceptibility of older individuals to muscle injury induced by ECC contractions [116]. Until now, there are no known effects that ECC training would have on muscle fatigue or diminished energy reserves that may be seen in some post-COVID-19 patients.

Because of the differences in cardiorespiratory and muscular stress, evaluation of the effectiveness of ECC exercise should combine a measure of intensity as well as a measure of DOMS. DOMS can be evaluated by a body diagram to identify the region and by a visual analogue scale to indicate its severity. The DOMS assessment should be combined with *rating of perceived exertion* (RPE) based on Borg scale, which has been shown to be a valid indicator of exercise intensity [117]. Borg will be more meaningful than VO_2_ or HR because of the lower cardiorespiratory stimulus (described above) to induce muscular changes from ECC compared to CONC training and thus, the rationale for matching RPE [15] when comparing intensities [118,119].

An interesting aspect to consider regarding including ECC in the PR programs is the best form to identify/recognize/determine the intensity of exercise in ECC training. Although there are no studies in post-COVID-19 patients, COPD patients’ data report that the perception of physical exertion (assessed according to the original or modified Borg scale) is key to determining the intensity of eccentric exercise, with a typical Borg value of 14 “*somewhat hard*” (original scale, 6 to 20) or 5 to 6 “*severe*” at “*very severe*” (modified Borg, 0 to 10). Thus, perceived exertion would commonly be above the target value of chosen physiological variables (e.g., heart rate, watts, gait speed, or % peak oxygen consumption) in concentric exercises. In other words, the intensity of eccentric exercise depends on the level of perceived exertion mentioned above; however, the physical workload can be increased considering the gradual process of effort adaptation to training [102,109,120,121].

**Figure 3:**
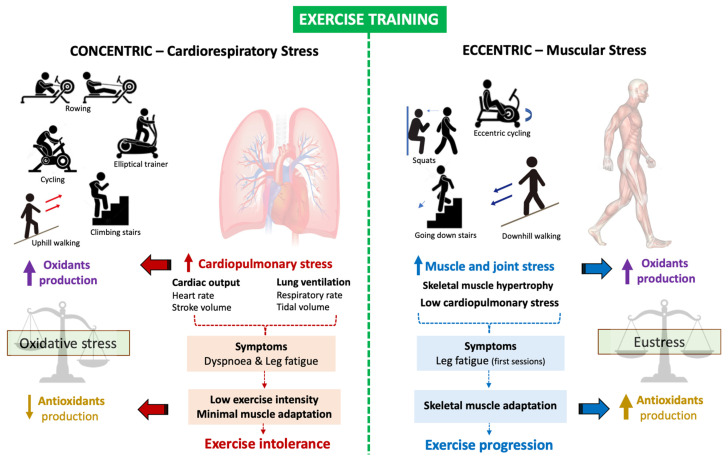
Overview of concentric and eccentric exercises (as the main components of the movement) on the cardiopulmonary and muscle systems, symptoms associated with these exercises, and their effects on redox balance.

To date, ECC training has been shown to be a well–tolerated exercise that induces improved functional capacity and locomotor muscle mass without inducing significant cardiopulmonary stress. Whether these adaptations are supported, in part, by training-induced decreases in inflammation and OS in post-COVID-19 patients or if this type of training is safe for them remains unknown. Figure 3 illustrates the impact of concentric and eccentric exercise training on body systems and their effect on redox balance.

## 9. Conclusions

Patients recovered from moderate-to-severe damage by ARDS associated with COVID-19 are left with deleterious functional impairments. Exercise has a crucial role in the post-discharge pulmonary rehabilitation. Although CONC exercises are safe and the conventional type of training, they induce exercise–limiting cardiopulmonary stress, dyspnoea, and fatigue. Hence, decreased tolerance and adherence to training can dramatically mitigate potential benefits. In contrast, ECC is a novel type of training often used by athletes but much less frequently in clinical contexts. Recent reports demonstrate significant increases in functional capacity and muscle mass with less dyspnoea and fatigue symptoms in ECC versus CONC training in COPD patients. However, data in outpatients post-COVID-19 is lacking. Thus, investigations are required that examine the effectiveness, tolerance, adherence, and functional improvements to PR programs induced by ECC on this population in comparison to CONC training.

## Data Availability

Not applicable.
